# Knowledge and Attitudes towards Epilepsy of Croatian General Student Population and Biomedical Students: A Cross-Sectional Study

**DOI:** 10.3390/healthcare11182550

**Published:** 2023-09-14

**Authors:** Tomislav Žuvela, Branka Filipović-Grčić, Doris Rušić, Dario Leskur, Darko Modun, Tin Čohadžić, Josipa Bukić, Ana Šešelja Perišin

**Affiliations:** Department of Pharmacy, University of Split School of Medicine, Šoltanska 2, 21000 Split, Croatia; tomislav.zuvela@mefst.hr (T.Ž.); brankafg@gmail.com (B.F.-G.); drusic@mefst.hr (D.R.); dmodun@mefst.hr (D.M.); tcohadzic@kbsplit.hr (T.Č.); jbukic@mefst.hr (J.B.); aperisin@mefst.hr (A.Š.P.)

**Keywords:** epilepsy, knowledge, attitudes, students, Croatia

## Abstract

Epilepsy causes a significant burden to patients as it is linked with various somatic and psychiatric comorbidities, social issues, impaired quality of life, and increased mortality. Improving the population’s knowledge and attitudes about epilepsy patients could be beneficial as it could raise social awareness and lead to more social support for patients. For those reasons, a survey-based cross-sectional study was conducted to determine Croatian students’ knowledge and attitudes toward epilepsy. A previously developed survey questionnaire was adapted for the Croatian setting and distributed online to the students (*n* = 544). Croatian students generally had positive attitudes towards people with epilepsy (median score 28.0, interquartile range 29.0–26.0, with the minimum possible score being 0.0 and the maximum 30.0), with the female gender (B (male) = 0.664 (95% CI −1.158, −0.170), *p* = 0.009), biomedical education (B (other) = −0.442, (95% CI −0.823, −0.061), *p* = 0.023), and personal experience in the form of witnessing the seizure (B = 0.519 (95% CI 0,098, 0.940), *p* = 0.016) as predictors of more favorable attitudes. Overall knowledge was satisfactory concerning most items, with the exception of first aid measures and risk factors. Educational intervention targeting bio-medical students and other students who might, in their future professional lives, be responsible for people suffering from epilepsy is needed to improve the gaps in their knowledge.

## 1. Introduction

Epilepsy is a chronic neurological disorder characterized by persistent seizures described as “sudden occurrences of transient signs and symptoms caused by abnormal and excessive or synchronous neuronal activity in the brain” [1]. Around 65 million people worldwide are considered to be affected. The disease causes a significant burden to the patients as it is linked with various somatic and psychiatric comorbidities, social issues caused by stigmatization, impaired quality of life, and increased mortality [2,3,4,5,6]. 

Patients with epilepsy have been misunderstood, discriminated against, and socially stigmatized for centuries. This, unfortunately, continues today and significantly affects the daily lives of patients [5,7]. Stigma is determined not only by the objective characteristics of the disease but also by social stereotypes created due to lack of information and unfounded fears [8]. Misconceptions and insufficient knowledge about epilepsy cause the development of negative attitudes toward patients with epilepsy and increase the stigmatization and psychosocial problems of patients [9]. An epileptic seizure is traumatic both for the patient and for those who witness it. It can be difficult for patients to face the reactions of others. Patients are afraid that a seizure in public will lead to condemnation and avoidance by other people. Therefore, epilepsy is not just a seizure. For the patient, the severity of the disease is determined not only by the severity of the disease symptoms but also by the numerous negative opinions about patients with epilepsy attributed to these patients by the members of society [9]. Stigma and discrimination negatively affect the quality of life and can often affect the patient less favorably than the seizures themselves [4,10]. Stigma stems from ignorance and misconceptions about epilepsy. Several studies have shown that more positive attitudes towards people with epilepsy were associated with a higher level of knowledge about epilepsy. Hence, strategies to combat social stigma include better education and marketing campaigns targeting the general population [11,12,13]. Patients who do not have enough knowledge about epilepsy have difficulties adjusting to and understanding their disease and, therefore, may stigmatize themselves, which, in addition to facing social stigma, adversely affects their lives [9,13]. Their fear of the reaction of their peers leads to social anxiety and isolation, which could worsen existing psychological distress [3,4,5,14]. Therefore, it was no surprise that epilepsy was linked to an increased suicide risk [2,15].

Previous studies have shown that knowledge and attitudes toward epilepsy in the general population are insufficient, as well as among teachers who were expected to be more educated and able to provide first aid [16,17]. Improving knowledge about epilepsy and their attitudes towards those affected could be beneficial as it could raise social awareness and increase social support for the patients [18]. Universities represent an environment that provides great opportunities for developing knowledge and building attitudes and beliefs based on scientific evidence, which could help young people create positive attitudes and correct behavior toward patients with epilepsy [19]. As students represent an educated group of society, it is important they have the right knowledge and attitudes about health issues [20]. Furthermore, it is especially important to evaluate the knowledge and attitudes of biomedical students as future medical workers who will directly work with epilepsy patients in the near future [21]. Misconceptions, prejudice, negative attitudes, and gaps in knowledge could influence the quality of the care the future healthcare workers will provide for their patients [22,23]. One multicentric study conducted on university health students in the United States, Portugal, South Africa, Argentina, and Brazil showed that an alarming percentage of biomedical students still have mistaken ideas about epilepsy. A third of the included participants thought epilepsy was a mental disorder, and almost half did not know the pathophysiology of the disease [20].

Epilepsy prevalence in Croatia has been previously determined to lie between 4.8–5.5/1000, with 53% of Croatian patients reporting feeling stigmatized [24,25]. A recent study showed that this prevalence might be underestimated, especially in less developed countries. Additionally, those poorer areas, with higher unemployment and lower education levels, might prove a further challenge for the patients, with more negative effects on their quality of life [26]. Furthermore, no studies assessing Croatian university students’ knowledge and attitudes have ever been published.

For the aforementioned reasons, we conducted a survey-based cross-sectional study to determine the Croatian students’ knowledge and attitudes towards epilepsy, with special emphasis on the biomedical students who were compared with the general student population to analyze how their education affected their results.

## 2. Materials and Methods

This cross-sectional survey-based study was conducted in July and August 2021. Participants were Croatian students enrolled in the academic year 2020/2021. The survey was conducted online as a Google Forms document, and participants from multiple universities in Croatia were able to participate. The survey was distributed through the official students’ administration offices of the faculties and through student representatives in Croatian student organizations. The study was approved by the University of Split School of Medicine Ethics Committee (approval number: 2181-198-03-04-21-0069), and participants had to give informed consent before accessing the survey questionnaire.

The questionnaire was developed by Mewes et al. [22], with additional items added from surveys by Alhalaiqa et al. [19] and Akça et al. [27]. It was adapted for Croatian students by first translating the adapted questionnaire into Croatian and then translating it back to English using native English speakers. The survey consisted of three parts and had a total of 26 items (Supplementary Materials). The first part gathered demographic data, such as enrolled study program, study year, age, and gender. It consisted of 11 items and included questions about personal and family history of epilepsy and personal experience with epilepsy. The second part consisted of 7 items and measured the knowledge about epilepsy. It included questions about epilepsy prevalence, symptoms, first aid measures, and activities that might pose a danger to people with epilepsy. The final part gathered the participants’ attitudes towards people with epilepsy and consisted of 6 items that measured the attitudes on a Likert scale (Definitely yes, Probably yes, Maybe, Probably no, and Definitely no) and 2 items where the choices were Yes/No/I do not know.

According to the Croatian Bureau of Statistics, 155,627 students were enrolled in the academic year 2020/2021 [28]. The sample size was calculated using the total number of students enrolled that year at all Croatian universities. With a confidence level set as 0.95 and a 5% margin of error, the necessary sample size was calculated and determined to be 384 participants.

Participants were grouped as either biomedical students or other. The biomedical field of studies was defined as one including the basic and clinical medical sciences, public health, veterinary medicine, dental medicine, and pharmacy, as defined by the National Council for Science, Higher Education and Technological Development, the government expert body that takes care of the development and quality of the scientific activity and the system of science, higher education, and technological development in the Republic of Croatia [29]. Data were presented as overall number and proportion (%), mean and 95% confidence interval (CI) or median and interquartile range (IQR), where applicable. Data were analyzed using the Chi-square test and Mann–Whitney U test. Each participant was assigned an attitude score based on their answers to questions regarding attitudes toward patients with epilepsy. The total score was a sum of answers, with the answer ‘Definitely yes’ being assigned five points, the answer on the opposite side of the Likert scale ‘Definitely no’ assigned one point, and answers in between assigned four, three, or two points. Higher scores indicated more favorable views towards people with epilepsy, and scores ranged from a minimum of six to a maximum of thirty points. Linear regression analysis determined the factors associated with more positive attitudes. Univariate analysis was performed for each variable, with the attitude score serving as a dependent variable. Multivariate regression analysis was further conducted by including factors significantly associated with attitude score in univariate analysis. Results were considered statistically significant for *p* < 0.05. Statistical analysis was conducted using SPSS (version 16.0, IBM Corporation, Chicago, IL, USA).

## 3. Results

### 3.1. Demographic Data

The number of students who started the survey was 595, with 51 not finishing the questionnaire. The completion rate was calculated to be 91.4%. A total of 544 students were included in the survey, of which 254 were biomedical students (46.7%). Students, on average, were 22.4 years old, with ages ranging from 18 to 40 years. Fifteen participants (2.8%) were diagnosed with epilepsy, while forty (7.4%) had a family member with epilepsy. More detailed information about the participants’ demographics is available in Table 1.

### 3.2. Results of Students’ Knowledge of Epilepsy

Only one participant stated that he never heard of epilepsy. A total of 333 (61.2%) students knew someone affected with epilepsy, and 155 (28.5%) had witnessed an epileptic attack. In total, 375 students (68.9%) correctly assumed it was possible to die from an epileptic seizure, while 145 (26.7%) stated they did not know the answer. Regarding that question, there was a significant difference between biomedical and other students as 76.4% of biomedical correctly guessed (and 17.7% stated they did not know the answer), while 62.4% of other students gave the correct answer (and 34.5% stated they did not know, *p* < 0.001). That not all seizures lead to a loss of consciousness was recognized by 307 students (56.4%), with a statistically significant difference between biomedical and other students (70.1% vs. 44.5%, *p* < 0.001). Around half of the students stated they did not know the prevalence of epilepsy (273, 50.2%), while 157 (28.9%) assumed the prevalence correctly. Epilepsy was recognized as a neurological disorder by 522 students (96.0%).

The number of participants who correctly attributed all the symptoms of epilepsy was 235 (43.2%), 150 of whom were biomedical students (61.2% of the total number), and 85 were other students (29.3% of the total number). Biomedical students were significantly better at recognizing the aforementioned symptoms, as seen in Table 2.

Regarding the possible first aid measures during the seizure, statistically significant differences were found between biomedical and other students in every item, as depicted in Table 3.

Comparable results were found between biomedical and other students regarding opinions as to how dangerous certain physical activities could be for patients with epilepsy (Table 4).

### 3.3. Results of Students’ Attitudes towards People with Epilepsy

A total of 44 students (8.1%) thought patients with epilepsy were more withdrawn than other people, with no statistically significant difference between biomedical and other students. Only 3 participants (0.6%) stated that patients with epilepsy had lower intelligence and abilities than the general population. Differences between biomedical and other students in attitudes towards people with epilepsy are shown in Table 5. Biomedical students had more positive attitudes towards women with epilepsy and their ability to have children, the only statistically significant difference between the two groups.

Participants’ attitude scores were high, with a mean value (standard deviation) of 27.6 (2.3) and a median value (interquartile range; minimum–maximum) of 28.0 (29.0–26.0; 18.0–30.0). The results of the linear regression analysis are shown in Table 6. Gender (*p* < 0.01), education (*p* < 0.05), and personally witnessing an epileptic seizure (*p* < 0.05) were associated with significantly different attitude scores in a univariate model. No significant association was found for the remaining factors. In the multiple regression model, female gender (*p* < 0.01), biomedical education (*p* < 0.05), and personally witnessing an epileptic seizure (*p* < 0.05) remained significantly associated with more positive attitudes towards patients with epilepsy.

## 4. Discussion

### 4.1. Knowledge of Epilepsy

Overall, the knowledge of Croatian students was satisfactory concerning most items, as most participants had heard of epilepsy, most correctly recognized it as a neurological disorder, and most knew the symptoms and certain first aid measures. As expected, biomedical students had better results than the general student population due to their education. However, there were certain gaps in knowledge that should be addressed. For example, most listed symptoms were recognized by students. However, every symptom was correctly recognized by little less than half of the total number of participants. Non-biomedical students were less adept in symptom recognition, with less than a third correctly assuming all the symptoms. The reason for this was that one symptom, the sudden unexpected behavior, was recognized by significantly fewer participants, which was also noted in a previous study [22]. Students were generally better at recognizing convulsions as a symptom, indicating they mostly equated epilepsy with generalized tonic-clonic seizures. In the meantime, biomedical students were significantly better at recognizing sudden unexpected behavior, a less commonly known symptom, which might indicate that they were more knowledgeable about different types of epilepsy due to the education they received. Another issue was first aid measures during the seizure. Inappropriate measures, such as holding the person down and putting objects into their mouths, were considered valid by 29.9% and 49.6% of biomedical students, while the proportions were even higher in the general student population. Administering rescue medication during the seizure was chosen by only 58.7% of biomedical and 50.7% of other students. While this measure might not always be necessary, it is of paramount importance that biomedical students know about this option. Also, other students should know that this measure exists as they might encounter people with epilepsy, especially if they start working with children, for example, in primary education [22]. Almost a quarter of biomedical students did not know it was possible to die from an epileptic seizure, an alarming result. The risk posed by certain leisure activities such as climbing, swimming, working out in the gym, and cycling were also severely underestimated. Again, the knowledge of the possibility of a fatal outcome and relevant risk factors is important for both biomedical students and other students who will come into contact with people with epilepsy during their professional lives [22,30]. Those misconceptions, particularly in the case of future healthcare workers, could adversely affect their practice in the future. Some previous research showed that caregivers and health professionals did not apply appropriate first-aid measures due to lapses in their knowledge. Consequently, there is a need for standardized training programs for both professionals and the public [31].

### 4.2. Attitudes towards People with Epilepsy

The attitudes of students of biomedicine, as well as other students, were generally positive. The vast majority of participants wanted to know if their colleague suffered from epilepsy. They would accept people with epilepsy as friends and would get romantically involved with them. Further, they had positive attitudes toward the employment of patients with epilepsy. Contrary to some former studies, Croatian students would disclose to their fellow students if they had epilepsy [22]. Participants generally believed women with epilepsy could give birth, with the biomedicine students having more positive answers, again likely due to being more knowledgeable about this matter. Favorable attitudes were further confirmed with the high attitude scores, as participants had a median score of 28 out of a maximum of 30. Biomedical students had higher attitude scores than the general student population, likely connected to their better knowledge, as noted in previous research [23,27]. In our study, female students displayed more favorable attitudes, which was in accordance with some previously published work [19,22] but in collision with the results of the study by Akca et al. [27]. Participants who witnessed an epileptic seizure also viewed epilepsy patients more favorably. Personal experience was a strong predictor of more positive attitudes in a previous study conducted on the general Croatian population [32], also noted in some previous research [33]. The same study found the high levels of tolerance towards people with epilepsy even higher than those of more developed countries [32], and our research confirmed that the same was true for the Croatian student population. Identifying and correcting the attitudes of the general population is desirable for improving social support for epilepsy patients. Patients with better social integration and support from their friends and family are more likely to engage in self-management routines and follow the health behavior recommendations that might improve their physical and social well-being [34,35].

### 4.3. Comparison of Results with Other Studies

The high knowledge of the Croatian biomedical student population was comparable to higher scores seen in other studies. Medical students from Palestine, Saudi Arabia, and Turkey, as expected, also had high knowledge scores [23,27,36,37,38]. A study conducted on German first- and second-year university students showed gaps in knowledge regarding first-aid measures and recognition of less common symptoms, comparable to the results of this study. However, medical students from the previously mentioned study did not have better knowledge or attitude scores when compared to non-biomedical students, which was a significant finding in this study [22]. The reason for the difference could be the inclusion of higher-year students in our study, who were taught about epilepsy in various subjects held in higher-year curriculums.

Previous research noted a directly proportional correlation between students’ knowledge about the disorder and their attitudes [21,36,39]. Contrary to those findings, one study on health management department students found no difference in attitudes between those with high and low knowledge about the disorder [40]. The present study did not directly correlate the knowledge and attitudes, but more knowledgeable biomedical students had more positive attitudes than non-biomedical students in our linear regression analysis.

### 4.4. Interventions for Knowledge Improvement

The most concerning finding in this study was the misconceptions about the first aid measures, particularly the misconceptions of the biomedical students. Knowledge about helping people having seizures was found to be insufficient among both students [22,27] and the general public [33,41,42,43] in the prior studies. Changes in biomedical students’ curriculums that would include more practical instructions on how to treat seizures might be beneficial to improve their knowledge retention and expertise. Furthermore, increasing the awareness about epilepsy and dispelling the misconception, especially about first aid measures, of the public is required. One of the possible solutions is seizure first aid training certification directed toward the general population [43]. Such programs might be excessive and unnecessary for everyone, but perhaps such training should be given to people who might find themselves in the care of epilepsy patients [22,42]. Educational campaigns targeting primary school teachers improved their responses to seizures and helped raise epilepsy awareness [42]. A similar study conducted in Turkey and Nigeria also showed improvements in teachers’ acceptance and perceptions of the children suffering from epilepsy and first aid measures after a brief education [30,44]. Certain authors suggested including educational courses in the school curriculum to reduce the stigma and teach schoolchildren basic first aid measures. [10,45,46]. To be more efficient, such educational interventions could be expanded to include teachers of all educational levels, school auxiliary professionals, and parents. Further, participation in a seminar leads to more positive attitudes toward patients with epilepsy and better knowledge retention among fifth-year medical students [10]. Modern technologies were previously suggested as a tool to spread knowledge about first aid measures, for instance, the online simulation videos that could be shared on social networks [41].

### 4.5. Study Limitations

This study has certain limitations. It was designed as a cross-sectional study, which provided only an observation, and its purpose was not to show any causal relationship. Moreover, the survey was conducted online, which could limit the representability of the sample as it was necessary to have internet access to participate in the study. However, as the participants were young students, the population that should have sufficient digital literacy and available free internet access at their faculties, we believed the results were not adversely affected by this limitation. More limitations that could lead to sampling bias were using a convenient sample and voluntary participation, which could lead to overrepresentation by the students more interested in the subject. Another issue that could have introduced bias was the sampling of the subgroups, as no specific strategy to distribute the questionnaire among two distinct groups was executed. However, we believe the effects were partially alleviated with the large overall sample size. A further limitation was a significant gender difference, with more women participating in the study. In Croatia, more women than men pursue university degrees, especially in the biomedical field, of which students made up almost half of our sample. However, differences in gender representation in this study could still influence the results.

## 5. Conclusions

Croatian students did not show prejudice toward patients with epilepsy, with female gender, and personal experience in the form of witnessing the seizure as predictors of more favorable attitudes. Biomedical students showed better knowledge and more favorable attitudes towards those affected with the disease than the general student population, likely due to directly receiving education concerning epilepsy. Generally, the level of knowledge was acceptable for most items, except for first aid measures and risk factors. There is a need for educational interventions aimed at biomedical students and other students who might be responsible for people suffering from epilepsy during their professional lives to address and enhance their knowledge gaps. Additional research is required to gain a more comprehensive understanding of the specific challenges and factors influencing knowledge gaps in these areas. Knowledge from our and upcoming studies could lead to more targeted and effective educational interventions in the future.

## Figures and Tables

**Table 1:** Demographic characteristics of the participants.

Characteristic	*n* (%)
Faculty	
Medicine	104 (19.1)
Dental medicine	82 (15.1)
Pharmacy	68 (12.5)
Science	110 (20.2)
Civil Engineering, Architecture and Geodesy	43 (7.9)
Economics, Business and Tourism	39 (7.2)
Other	98 (18.0)
Gender	
Male	98 (18.0)
Female	446 (82.0)
Study year	
1st	106 (19.5)
2nd	86 (15.8)
3rd	118 (21.7)
4th	106 (19.5)
5th	87 (16.0)
6th	41 (7.5)
Personal diagnosis of epilepsy	
No	529 (97.2)
Yes	15 (2.8)
Family diagnosis of epilepsy	
No	504 (92.6)
Yes	40 (7.4)
Family member a healthcare worker	
No	415 (76.3)
Yes	129 (23.7)
Age	
Mean (standard deviation)	22.4 (2.6) years
Range	18–40 years

**Table 2:** Differences between biomedical and other students in epilepsy’s symptoms recognition. Results defined as the number of participants who recognized the symptoms.

Symptom	Biomedicine and Health Students *n* (%)	Other Students *n* (%)	*p* *
Sudden unexpected behavior	172 (66.7)	124 (43.4)	<0.001
Facial muscles’ spasms	238 (92.2)	244 (85.3)	0.035
Short loss of consciousness	245 (95.0)	248 (86.7)	0.004
Convulsions of the extremities	249 (96.5)	250 (87.4)	0.001
Convulsions of the whole body	247 (95.7)	262 (91.6)	0.135

* Chi-square test.

**Table 3:** Differences between biomedical and other students’ knowledge of first aid measures in case of a seizure. Results defined as the number of participants who thought a measure was appropriate.

Measure	Biomedicine and Health Students *n* (%)	Other Students *n* (%)	*p* *
Administration of appropriate medication, if available	149 (58.7)	147 (50.7)	0.006
Turn the person on its’ side	229 (90.2)	237 (81.7)	0.029
Put something soft under the head	229 (90.2)	203 (70.0)	<0.001
Hold the person firmly on the ground to prevent movement	76 (29.9)	112 (38.6)	<0.001
Put the solid object in the mouth to prevent an injury to the tongue	126 (49.6)	166 (57.2)	<0.001
Immediately call an ambulance	233 (91.7)	258 (89.0)	0.036

* Chi-square test.

**Table 4:** Differences between biomedical and other students regarding opinions as to how dangerous physical activities are to patients with epilepsy. Results were defined as the number of participants who assumed the respective activity was more dangerous for people with epilepsy.

Activity	Biomedicine and Health Students *n* (%)	Other Students *n* (%)	*p* *
Clubbing	234 (92.1)	252 (86.9)	0.501
Cycling	63 (24.8)	52 (17.9)	0.196
Swimming	85 (33.5)	77 (26.6)	0.139
Climbing	79 (31.1)	80 (27.6)	0.787
Exercising in the gym	71 (27.9)	65 (22.4)	0.053

* Chi-square test.

**Table 5:** Differences between biomedical and other students in attitudes towards people with epilepsy. Results are defined as a median value of agreement with the statement on a Likert scale, with 5 signifying Definitely yes and 1 signifying Definitely no.

Statement	Biomedicine and Health Students *	Other Students *	*p* ^#^
I would want to know if a fellow student suffered from epilepsy	5 (5-4)	5 (5-4)	0.637
I would tell my fellow students if I had epilepsy	4 (5-4)	4 (5-4)	0.696
I would welcome someone with epilepsy into my circle of friends	5 (5-5)	5 (5-5)	0.228
I would go on a romantic date with someone with epilepsy	5 (5-5)	5 (5-4)	0.174
Women with epilepsy can give birth	5 (5-5)	5 (5-4)	<0.001
I would hire a person with epilepsy if he/she had appropriate qualifications	5 (5-5)	5 (5-5)	0.195

* Median (Interquartile range); ^#^ Mann Whitney U test.

**Table 6:** Linear regression derived estimates and 95% CI with dependent variable defined as a score indicating attitude towards epilepsy patients.

Characteristics	Univariate Analysis, Estimate 95% CI	Multivariate Analysis, Estimate 95% CI
Gender		
Female	Reference	
Male	−0.677 (−1.175, −0.179) **	−0.664 (−1.158, −0.170) **
Education		
Biomedical	Reference	
Other	−0.441 (−0.825, −0.057) *	−0.442 (−0.823, −0.061) *
Study year		
1st	−0.114 (−0.765, 0.537)	
2nd	−0.062 (−0.746, 0.623)	
3rd	−0.190 (−0.826, 0.446)	#
4th	−0.369 (−1.020, 0.282)	
5th	Reference	
6th	−0.140 (−0.713, 0.992)	
Family diagnosis of epilepsy		
No	Reference	#
Yes	0.237 (−0.501, 0.975)	
Family member a healthcare worker		
No	Reference	#
Yes	0.248 (−0.205, 0.700)	
Are you acquainted with someone affected by epilepsy?		
No	Reference	#
Yes	0.334 (−0.060, 0.728)	
Have you ever witnessed an epileptic seizure?		
No	Reference	Reference
Yes	0.505 (0.080, 0.930) *	0.519 (0.098, 0.940) *

* *p* < 0.05, ** *p* < 0.01, # multivariate regression analysis was not conducted for factors not significantly associated with attitude score in univariate analysis.

## Data Availability

The datasets used and analyzed during this study are available from the corresponding author upon reasonable request.

## Supplementary Materials

The following supporting information can be downloaded at: https://www.mdpi.com/article/10.3390/healthcare11182550/s1.
