# Supplementary material for: Knowledge and Attitudes towards Epilepsy of Croatian General Student Population and Biomedical Students: A Cross-Sectional Study
Source: Healthcare (Basel). 2023 Sep 14;11(18):2550. doi: 10.3390/healthcare11182550 (PMC10531231; doi:10.3390/healthcare11182550)
Supplement: Supplementary file 1 [file healthcare-11-02550-s001.zip › healthcare-2591714-supplementary.pdf]

Supplementary file

Questionnaire

Faculty

- Medicine
- Dental medicine
- Pharmacy
- Science
- Civil Engineering, Architecture and Geodesy
- Economics, Business and Tourism
- Other

Sex

- Male
- Female

Age: ______ years

Study year

- 1st
- 2nd
- 3rd
- 4th
- 5th
- 6th

Do you have epilepsy:

- No
- Yes

Do you have a relative with epilepsy?

- No
- Yes

7. Is any member of your immediate family a healthcare worker?

- No

- Yes

8. If the answer of the previous question is yes, please select or write his exact profession:

- Physician

- Dentist

- Pharmacist

- Nurse

- Other: __________

9. Have you ever heard about epilepsy?

- No

- Yes

10. Are you acquainted with someone affected by epilepsy?

- No

- Yes

11. Have you ever witnessed an epileptic seizure?

- No

- Yes

12. Is it possible to die from an epileptic seizure

- No
- Yes
- I do not know

13. Everyone suffering from epilepsy experiences a loss of consciousness during a seizure.

- No
- Yes
- I do not know

14. Prevalence of epilepsy in general population is approximately:

- 0.01%
- 0.1%
- 1%
- more than 10%
- I do not know

15. Epilepsy is:

- Infectious disease
- Psychiatric disease
- Form of mental retardation
- Neurological disease

16. Are the following activities more dangerous for people with epilepsy?

**Clubbing**	Yes	No	I do not know
**Cycling**	Yes	No	I do not know
**Swimming**	Yes	No	I do not know
**Climbing**	Yes	No	I do not know
**Exercising in the gym**	Yes	No	I do not know

17. Which of the following symptoms are linked to a seizure?

**Sudden unexpected behaviour**	Yes	No	I do not know
**Facial muscles' spasms**	Yes	No	I do not know
**Short loss of consciousness**	Yes	No	I do not know

Convulsions of the extremities Yes No I do not know

Convulsions of the whole body Yes No I do not know

18. Possible measures that should be taken during an acute seizure are:

Administration of appropriate medication, if available

Yes No I do not know

Turn the person on its' side

Yes No I do not know

Put something soft under the head

Yes No I do not know

Hold the person firmly on the ground to prevent movement

Yes No I do not know

Put the solid object in the mouth to prevent an injury of the tongue

Yes No I do not know

Immediately call an ambulance

Yes No I do not know

19. People with epilepsy are not more withdrawn than other people.

Yes No I do not know

20. Patients with epilepsy have lower intelligence and abilities.

Yes No I do not know

21. I would want to know if a fellow student suffered from epilepsy

Definitely yes

Probably yes

Maybe

Probably no

Definitely no

22. I would tell my fellow students if I had epilepsy

Definitely yes

Probably yes

Maybe

Probably no

Definitely no

23. I would welcome someone with epilepsy into my circle of friends

Definitely yes

Probably yes

Maybe

Probably no

Definitely no

24. I would go on a romantic date with someone with epilepsy

Definitely yes

Probably yes

Maybe

Probably no

Definitely no

25. Women with epilepsy can give birth

Definitely yes

Probably yes

Maybe

Probably no

Definitely no

26. I would hire a person with epilepsy, if he/she had appropriate qualifications

Definitely yes

Probably yes

Maybe

Probably no

Definitely no
